# The Social Brain Is Not Enough: On the Importance of the Ecological Brain for the Origin of Language

**DOI:** 10.3389/fpsyg.2016.01138

**Published:** 2016-08-02

**Authors:** Francesco Ferretti

**Affiliations:** Department of Philosophy, Communication and Performing Arts, Roma Tre UniversityRome, Italy

**Keywords:** ecological brain, global coherence, mental time travel, narrative, origin of language, storytelling, relevance theory, social brain

## Abstract

In this paper, I assume that the study of the origin of language is strictly connected to the analysis of the traits that distinguish human language from animal communication. Usually, human language is said to be unique in the animal kingdom because it enables and/or requires intentionality or mindreading. By emphasizing the importance of mindreading, the social brain hypothesis has provided major insights within the origin of language debate. However, as studies on non-human primates have demonstrated that intentional forms of communication are already present in these species to a greater or lesser extent, I maintain that the social brain is a necessary but not a sufficient condition to explain the uniqueness of language. In this paper, I suggest that the distinctive feature of human communication resides in the ability to tell stories, and that the origin of language should be traced with respect to the capacity to produce discourses, rather than phrases or words. As narrative requires the ability to link events distant from one another in space and time, my proposal is that in order to explain the origin of language, we need to appeal to both the social brain and the ecological brain – that is, the cognitive devices which allow us to mentally travel in space and time.

## Introduction

In this paper, I argue that the study of the origin of language is strictly connected to the analysis of the traits that distinguish human language from animal communication. The basic assumption of my proposal is that the distinctive feature of language resides in the ability, typically human, to tell stories, and that the origin of language should be traced in reference to this ability to produce discourses, rather than phrases or words. Supporting the narrative basis of language has important effects both in terms of the recognition of the essential properties of human communication and the investigation of the cognitive systems dedicated to the processing of such properties. Since global coherence is the foundational property of the ability to produce and understand discourses, this paper analyzes the cognitive systems that can process such a property.

The study of the cognitive systems that process the narrative level is a privileged point of view to test one of the most relevant cognitive theories about the origin of language: the idea that the transition to language from animal communication has to be found in the specific social brain of our species. Against such idea, my thesis is that the cognitive devices of the social brain are unable to guarantee the narrative character of human communication, and that the properties processed by the social brain are not sufficient to justify the origin of human language. In order to explain the origin of language, in fact, behind the social brain we have to appeal to the ecological brain (i.e., the cognitive devices at the base of our ability to navigate in space and time). The first move to examine the specific role played by the ecological brain in the origin of language is to justify the narrative character of human communication.

## Narrative Representation

The reason why we should consider the ability to tell stories as the distinctive trait of language lies in the fact that narrative is the specific way through which humans represent their experiences. Following Bruner (1991, p. 4), in fact, “we organize our experience and our memory of human happenings mainly in the form of narratives — stories, excuses, myths, reasons for doing and not doing, and so on”. Despite being in contrast with Bruner’s cultural constructivism, the idea that narrative is the distinctive trait of human experience representation serves as the basis of my paper’s assumptions as well.

The hypothesis that human nature is defined in reference to narrative abilities has received consensus in the scientific community (Thompson, 2010; Corballis, 2014, 2015; McBride, 2014). Niles (1999), for example, defined humans in terms of *Homo narrans*. Maintaining that “humans (…) *are* unique in sharing stories”, Thompson (2010, p. 398) suggested that the individuals of our species are better defined as *Homo fabulator*. Corballis (2015, p. 107) proposed a quite similar definition:

If there is anything that defines our species as unique (…) it is the telling of stories, and the invention of language as the means of doing so. (…) Other animals, even rats, may well undertake limited mental travels through limited domains, but stories allow us to expand our mental lives to unlimited horizons.

Following such considerations, it is plausible to maintain that the human ability to tell story might be considered as a product of biological evolution process. According to Gottschall (2012), for example, storytelling capacities play an important adaptive role. Furthermore, in his view, the narrative ability works very similarly to a flight simulator: it allows humans to gain experience of the most intricate affairs of life by sitting safely in an armchair. Nowadays, the topic of the narrative evolution remains the focus of a very interesting debate (Dautenhahn, 2002; Hirstein, 2005; Gottschall and Wilson, 2005; Scalise Sugiyama, 2005; Collins, 2008, 2013; Boyd, 2009; Gottschall, 2012; Herman, 2013).

In this paper, I maintain that the question of language origin has to appeal to the analysis of the abilities to tell stories because in storytelling nestle the properties that have allowed humans to develop a very different kind of communication from that used by other animals. The justification of my thesis goes through a two-step argument: to prove that narrative abilities precede (logically and temporally) language and to prove that language was born specifically in order to tell stories. Such a two-step argument will receive support from empirical research on the cognitive architectures implicated in narrative processing. And it is in reference to such an analysis that it will be possible to appreciate the role played by the navigational systems in space and time (two of the main components of the ecological brain) in order to explain the narrative hypothesis of the origin of language. Before analyzing the cognitive system implicated in narrative processing, it is right to deal with the hypothesis (the prevalent one) that the social brain is the necessary and sufficient condition to explain the transition from animal to human communication.

## The Social Origin of Language

The social nature of language is a well-known and not disputable fact. In cognitive science, a lot of models explain the origin of language calling upon the processing systems in use in the management of social behavior (Origgi and Sperber, 2000; Sperber, 2000; Tomasello, 2008; Scott-Phillips, 2014, 2015). In this perspective, the characteristic features of human communication depend upon the properties processed by the cognitive devices that compose the social brain.

### The Social Brain

Frith and Frith (2010, p. 165) maintained that the social brain allows “humans to boldly go where no other species has been”. In order to understand the kind of relationship that ties language and social brain together it is necessary to clarify the specific cognitive function of the social brain. In a paper that represents a cornerstone in the study of the social life of primates from a cognitive point of view, Humphrey (1976, p. 307) maintained that the brain evolution of the great primates is not dependent on the challenges that originate from the ecological world. By observing mountain gorillas’ behaviors, Humphrey theorized that the main cognitive abilities of great primates are primarily dependent on the management of the social worlds, as “the chief role of creative intellect is to hold society together”. The same considerations are applicable to the case of human beings: the creativity and flexibility in characterizing the behaviors of the individuals of our species may be considered a form of adaptation to the challenges imposed by the social environment. In a similar way, Dunbar (1998, 2009) maintained that the management of social relationships has led humans to the development of a brain that is unique in the animal world. The role of the nervous system in the management of relations with other individuals emerges clearly when, following Berthoz (2002), the brain is considered as a machine essentially assigned to predict future, to anticipate the consequences of one’s own or other people’s action, and to “buy time”. The difference between the solution of problems imposed by the physical environment and competition with the other competing individuals for the solution of the same problems is crucial in adaptation. With the expression “Machiavellian intelligence,” Byrne and Whiten (1988) referred to one of the most pressing evolutionary challenges with which primates had to deal, namely, the ability to predict and control the behavior of the others, using them as a means to their own ends. It is neither the case to delve into the details of evolutionary genesis of the social brain, nor to discuss in detail the social intellect specific functions. As my main interest in this section is the analysis of the relationship between the social brain and the origin of language, a tangible clue to start is the distinction between a communication founded on involuntary signals and a communication founded in voluntary signals.

Not always would communication have a voluntary character. The social brain is able to grasp the involuntary signals that an organism produces by simply moving into the environment. Frith and Frith (2010) maintained that from a biological motion the social brain is able to obtain information related to the intentional states of an individual and to identify the causes of his actions before he acts effectively. The most interesting case to study the relationship between the social brain and language is the case of voluntary communication. Referring to the voluntary acts of communication, it is possible to construct a specific hypothesis of the origin of language. Frith and Frith maintained that such acts have an ostensive character, a feature of great importance, as we shall see, to identify the essential features of human language. The reference to the ostensive character of communication leads us to one of the main theoretical framework to study the role of social brain in the origin of language: the Relevance Theory (RT) proposed by Sperber and Wilson (1986).

### Ostensive Communication

At the foundation of RT is the criticism to the code model of communication (Shannon, 1948). Against such a model, Sperber and Wilson (1986) appealed to the theory of meaning founded on speaker’s intentions proposed by Grice (1957, 1969, 1982). Unlike in animal communication (due to the model code), in human communication, what the speaker says will not always coincide with what is intended to mean. The shifting focus of attention from what the speaker says to what he/she intends to say has profound implications on the level of cognitive architectures: as pragmatic interpretation is ultimately an exercise in metapsychology, human communication implies a mindreading device (Sperber and Wilson, 2002). This conception has implications also for the origin of language: If the transition to human communication has to appeal to the meta-representational plan, then a mindreading system has to be a cognitive prerequisite for the advent of language (Origgi and Sperber, 2000). In Sperber’s opinion:

[H]uman communication is a by-product of human meta-representational capacities. The ability to perform sophisticated inferences about each other’s states of mind evolved in our ancestors as a means of understanding and predicting each other’s behavior. (…) Language made inferential communication immensely more effective. It did not change its character. All human communication, linguistic or non-linguistic, is essentially inferential. (Sperber, 1995, p. 199).

That said, the reference to the speaker’s intentions and to the mindreading system are too general to provide an account of the specific nature of human communication. To understand correctly the questions involved in the origin of language, it is necessary to propose more articulated distinctions.

At the level of properties, we have to distinguish between the informative and the communicative character of the speaker’s intention. Sperber and Wilson (2002, p. 255) schematized in this way the two forms of intention at the basis of ostensive communication:

#### The Informative Intention

The intention to inform an audience of something;

#### The Communicative Intention

The intention to inform the audience of one’s informative intention

If, during a dinner at the restaurant, looking for the gaze of the waiter, I lifted the empty bottle of wine stretching out my arm, the waiter would understand my request to have a new bottle (full, of course). Bringing a new bottle of wine, the waiter would show that he had caught my informative intention and thus that he understood the content of my request. For communication to be successful, however, in addition to my informative intention, there is something further that the waiter must have caught. Through eye contact and the attention to my arm gesture, the waiter has to catch my communicative intention, that is, the intention to inform him of my intention to have another wine bottle. What kind of cognitive systems ensures the development of the two different forms of intentions?

Ostensive communication gains support on the idea that the specific nature of human communication “depends upon sophisticated forms of social cognition that are unique to humans and which evolved in our species as a result of our über-social nature” (Scott-Phillips, 2014, p. xiii). If the specific trait of human communication has to be referred to communicative intentions, then the appeal to a general metacognitive system is not a sufficient move to explain the ostensive character of communication. What is needed further is a meta-communicative system, a sub-module of the mind-reading module, an automatic application of a relevance-based procedure to ostensive stimuli, and in particular to linguistic utterances (Sperber and Wilson, 2002). I will return soon to the role that this submodule plays on the origin of language but before that, a consideration of a more general character.

According to the proponents of RT, the origin of language is closely related to the transition from the code model to ostensive communication. As recent studies have confirmed the intentional nature of some forms of animal communication (Crockford et al., 2012; Schel et al., 2013) an important point to stress here is that the intentional nature of communication is insufficient to ensure this form of transition. In Scott-Phillips’s (2014, p. 4) opinion, in fact, ostensive communication

is not only intentional, it is *overtly* intentional. In other words, not only signal used in a voluntary (i.e., intentional) way, but this fact is made explicit (overt) to the audience, and this explicitness contributes to successful comprehension. Consequently demonstration of intentionality in animal communication is not sufficient to demonstrate meaning in the sense that is relevant to comparisons with human communication.

The result of these arguments is that if ostensive communication cannot be reduced to the intentional character, the origin of human communication must relate to the emergence of a kind of features that is radically new with respect to the features present in other forms of animal communication. In Scott-Phillips’ hypothesis “nothing that looks even remotely like language can emerge prior to the evolution of ostensive-inferential communication” (Scott-Phillips, 2014, p. 46). Now, is ostensive communication really the exclusive prerogative of human beings? Are the individuals of our species really disposed to the cognitive characteristics requested by ostensive communication? The answer to these questions is related to the animal mindreading debate.

### Animal Mindreading

The starting point of the debate is represented by Premack and Woodruff’s (1978) question posed in their famous paper: *Does the chimpanzee have a “Theory of Mind”?* As is known, the authors answered positively to the question in this paper. Ten years after the work written with Woodruff, however, Premack (1988) returned to the topic and proposed a more cautious version of mindreading abilities of great apes. In his new study, he was willing to recognize that apes have a weaker theory of mind than that of humans: chimpanzees would be able to attribute perceptual and volitional states but would not be able to attribute epistemic states (intentions and beliefs) to others. Also, Call and Tomasello (2008) espoused a similar position; their opinion is that it is possible to assign a broad form of mindreading to chimpanzees, while it is not correct to attribute a narrow form of mindreading to them:

It is time for humans to quit thinking that their nearest primate relatives only read and react to overt behavior. (…) Even if chimpanzees do not understand false beliefs, they clearly do not just perceive the surface behavior of others and learn mindless behavioral rules as a result. (…) In a broad construal of the phrase “theory of mind,” then, the answer to Premack and Woodruff’ pregnant question of 30 years ago is a definite yes, chimpanzees do have a theory of mind. But chimpanzees probably do not understand others in terms of a fully human-like belief-desire psychology in which they appreciate that others have mental representations of the world that drive their action even when those do not correspond to reality. And so in a more narrow definition of theory of mind as an understanding of false beliefs, the answer to Premack and Woodruff’s question might be no, they do not (Call and Tomasello, 2008, p. 191).

Distinguishing between mindreading abilities between a broad and a narrow sense is an important first step to address the issue properly if apes are capable of attributing intentional states, namely, to say that the question must be interpreted in terms of a continuum that does not imply absolute answers in terms of yes or no is the right way to proceed.

Having said that, what would the results of studies on mindreading in non-human animals tell us on the origin of language? Not much, for the advocates of the ostensive model. Following Scott-Phillips (2014, p. 97), in fact, since the mental state involved in ostensive communication are epistemic states such as “intentions and beliefs (a “belief-desire psychology”), and not simply knowledge states”, and since empirical studies have prompted us to exclude that chimpanzees are capable of mental attribution of epistemic states, it follows that chimpanzees lack the cognitive prerequisites to develop an ostensive communication.

The argument of Scott-Phillips is not fully convincing. The reference to the type of state of mind does not seem to be the point at issue to explain the ostensive character of human communication. In fact, it is unclear whether the distinction between types of mental states would be considered the right move to do in order to distinguish between informative and communicative intention. What is required to grasp the overtly intentional nature of ostensive communication is a type of skills independent from the state of mind to which it applies. What we really need is a requirement easier to comply: the ability to call attention to the fact (e.g., as when we use eye contact to ask the waiter for a new bottle of wine) that we intend to communicate something to someone. In light of these considerations, the possibility of attributing ostensive communication to non-human animals deserves further study.

### A Deflationary Proposal

As Moore (2016, p. 225) maintained, “consensus is that—at least in humans—where eye contact is used to address an utterance to its intended audience, this suffices to make communicative behaviors ostensive”, eye contact represents a useful tool for evaluating the ostensive capacity of non-human animals. The question of whether these animals are able to use eye contact to the communicative purposes is controversial. Referring to Call et al. (2000) empirical data, Scott-Phillips was skeptical; Moore (2016; see also Csibra, 2010) maintained the opposite perspective: In his view, the empirical results demonstrate that chimpanzees seek eye contact before producing gestures (Hostetter et al., 2001; Povinelli et al., 2003). Beyond the behavioral criteria, Moore (2016) also discussed the psychological criteria for ostension. He criticized the thesis that ostensive communication must appeal to sophisticated systems of social cognition. His idea (see also Gómez, 1994, 2007) is that:

It is sufficient for acting with communicative intent that one produce (sincerely and in conjunction with one another) a *sign* in order to elicit some behavioral response or action r from an interlocutor and an *act of address*, with which one directs one’s performance of that sign to the attention of one’s interlocutor. (Moore, 2016, p. 226).

Adhering to a deflationary proposal implies that the ability to produce ostensive communication goes beyond the limits of the individuals of our species. Studies that demonstrated chimpanzees’ ability to deliberately solicit the attention of others before gesturing evinced that these animals are capable of ostensive communication “even if the messages they produce were only even very simple ones, and even if they struggled to interpret many human forms of communication” (Moore, 2016, p. 227). These arguments can be used to maintain that the ostensive character of the communication does not depend on the type of mental state or on the complexity of the representational level.

The conclusion drawn from such arguments is that the adhesion to the deflationary approach comports a weakening of the thesis of the existence of a property able to distinguish clearly between animal and human communication. This conclusion has important consequences for the evolution of language debate: As ostensive communication is not a useful criterion to distinguish human language, then ostensive communication is not a useful criterion to explain the origin of human communication. The social brain hypothesis is not strong enough a criterion for characterizing human language, and to find such a criterion we have to seek elsewhere. Before this exposition presents this new direction, one last clarification is necessary.

Arguing that the social brain is insufficient to provide an account of the distinctive features of human communication, I do not intend to deny the role of mindreading in characterizing human language. Nor do I intend to deny the role of ostensive communication (and more generally of RT) in the study of the functioning and origin of language. What I intend to criticize is the idea that, in order to characterize human language, it is sufficient to refer to a single principle and a single processing system. Ostensive communication is definitely a characteristic of human communication, but for the reasons that I have examined, it is not a robust criterion to distinguish language from animal communication.

The study of the ecological niche in which human communication have originated proved to be a useful way to establish a stronger criterion of differentiation. The analysis of the effective environment in which our ancestral precursors had experimented on the first forms of human communication paves the way to a different approach to the origin of language. It is from the analysis of this ecological niche, as I shall see, that comes to the fore the possibility to study the origin of language in relation to the ability to tell stories, the capacity that at the beginning of this paper I considered as the distinctive trait of human beings.

### Recruitment at the Origin of Language

According to Bickerton (2010), the selective pressures at the origin of language has to be referred to the type of social relationships (the recruitment) required by scavenging, the prevailing activity of food procurement in the Oldowan niche (Semaw et al., 2003; Plummer, 2004; Osvath and Gärdenfors, 2005). Since the exploitation of carcasses (especially the carcasses of megafauna, the richest source of food lays) was a risky activity, scavenging had been a selective pressure in favor of cooperation between individuals. Therefore, it is exactly in the activity of recruitment for cooperative purposes that our ancestral relatives have developed increasingly sophisticated forms of communication. As Bickerton (2010) maintained, the type of effective communication for the purposes of recruitment has to respect an important commitment: the need to provide detailed information about something that is not directly present at the audience’s view. A feature of this type is reflected in the recruitment strategies employed by animals very distant from us in evolution. After all, our ancestral relatives engaged in recruitment had behaved very similarly to how ants and bees behave when they inform their conspecifics about the location of the food. Fitch et al. (2010) provided empirical support to the hypothesis “that scavenged lifestyle selects for socio-cognitive and communicative abilities” (Fitch et al., 2010, p. 806).

The thesis that recruitment has to be considered as a selective pressure for the origin of language is in line with the social brain hypothesis and those theoretical models that consider social practices as an essential trait of human communication (Deacon, 1997; Christiansen and Chater, 2008; Tomasello, 2008). That the social brain plays a role in the origin of language, as I have already pointed out, is not obviously under discussion, the relevant question here is whether the social brain represents a sufficient condition for the origin of language. The case of recruitment will help us to clarify this point at issue.

Considering it more carefully, recruitment is not only an example in favor of the role of the social brain in the origin of language. For example, in the case of bees, it is true that communication between these insects “is devoted almost entirely to recruitment” (Bickerton, 2010, p. 208), but it is also true that in order “to continue searching for adequate “crowd” before themselves returning to the carcass [informants] would have had to give some indication as to where and/or how far away the carcass was located”. The case of bees provides a starting point to reflect on the connection between the social and the ecological brain in communication. The irruption of the ecological brain in social communication, as I shall see in detail, plays an important role in the revision of the language origin model based on the ostensive character of human communication. It refers to the ability to travel in space and time that will possibly explain our ability to tell stories: the typically human way of representing reality that underlies the uniquely human way of communicating. In order to specify the role of the ecological brain in the narrative origin of language, we have to discuss what makes narrative a narrative representation and what makes narrative representation a precondition of human communication.

## The Ecological Brain

What makes narrative representation so different from any other form of representation is primarily its temporal character. In Bruner’s (1991, p. 6) opinion:

A narrative is an account of events occurring over time. It is irreducibly durative. It may be characterizable in seemingly non-temporal terms (…), but such terms only summarize what are quintessential patterns of events occurring over time. The time involved, moreover, as Paul Ricoeur has noted, is “human time” rather than abstract of “clock” time. It is time whose significant is given by the meaning assigned to events within its compass.

Following Bruner, Dautenhahn (2002, p. 107) maintained that narrative allows humans to extend their temporal horizon: it is the ability to travel forward and back in time that makes narrative “fundamentally different from communicative non-narrative events that are limited to the immediate present”. A second aspect of narrative, strictly related to the temporal character, concerns the structure of causal connections between events. As Herman (2013, p. 237) affirmed “from a structural standpoint, one of the hallmarks of narrative is [the] linking of phenomenon into causal-chronological wholes”. Essentially, it is in the force of the causal and temporal structures characterizing the ability to storytelling that narrative “constitutes a primary resource for configuring circumstances and events into more or less coherent scenarios involving the experiences of persons” (ibid, p. 74).

That being said, some clarification is needed. While I recognize the validity of Bruner’s narrative representation analysis, in this paper, I propose an idea of the causal relationship between narrative and cognition that specularly respect the opinions of Bruner. From my point of view, it is cognition that constitutes narrative more than the contrary: the center of my hypothesis is the idea that “global coherence” (the property at the foundation of narrative) depends on the cognitive systems’ ability to project individuals in time and space and to process the structure of the causal links between events. Before entering in the analysis of the ecological brain’s role in the processing of language’s narrative structure, it is useful to clarify what kind of property is global coherence.

### Global Coherence

As the most heated discussions regarding the definition of global coherence relates to the question of its irreducibility to cohesion (cf. e.g., Giora, 1985; Adornetti, 2015), for my purposes in this paper, the most important issue concerns the relationship between global coherence and relevance. It is in reference to relevance that the question whether the social brain is able to guarantee the origin of language. In fact, if coherence is reducible to relevance, then, mindreading is able to govern the development of narrative, and the social brain can therefore be considered a sufficient condition for the origin of language; if, on the contrary, global coherence were not reducible to relevance, then mindreading would be unable to guarantee narrative processing, and the social brain would not be considered the cognitive system able to explain the origin of language.

Giora (1997, 1998) brought convincing arguments to the thesis of the irreducibility of global coherence to the principle of relevance. Such arguments have given rise to a heated debate with Wilson (1998). The debate’s focus concerns Giora’s thesis that narrative processing relies on the identification of the causal links governing the relationship between segments of discourse. However, this is not the case to delve into the details of the debate between Giora and Wilson. The issue that has to be highlighted for my purposes is that the argument used by Wilson (1998) in favor of the reducibility of coherence to relevance rests on the idea that all you need to process the narrative plan is a system that is able to grasp the speaker’s communicative intention independently through the speaker’s organization of the discourse segments in the communication flow. As Wilson (1998, p. 67) maintained what essentially enables us to understand a discourse “is the fact that it has an interpretation consistent with the principle of relevance, *whether or not the discourse segments are related*”, emphasis added). If Wilson’s thesis were correct, the social brain would be sufficient to explain not only the language functioning but also its narrative origin. That mindreading has a role in narrative is a known and not a controversial fact (cf. Mar, 2011): To be controversial is the fact whether mindreading can be considered the only system we need to explain the processing of global coherence. As it is clear from Wilson’s quote, the point in discussion to resolve the controversy is to decide if the causal relationship between the discourse segments plays a role in narrative processing.

Trabasso et al. (1984), Trabasso and Sperry (1985), and Trabasso and van den Broek (1985) revealed evidence in favor of the idea that the comprehension of global coherence implies the processing of the causal links between events. From the point of view of The Causal Network Model (CNM), the narrative structure of a discourse is represented by a causal network of events and event relations (Trabasso and Sperry, 1985). Moreover, the idea that narrative depends on the ability to manage the causal connections between events is highlighted in several studies on storytelling in individuals with Autism Spectrum Disorders (ASDs) (Losh and Capps, 2003; King et al., 2013, 2014; Sah and Torng, 2015). Subjects with ASD “appeared to have difficulty representing and retelling the gist of stories” (Diehl et al., 2006, p. 88). Since such difficulty is connected to the “difficulty [of] putting story retellings together as a meaningful chain of events”, it has been proposed that the deficiencies in global coherence of individuals with ASD could be explained in reference to their difficulty in identifying the causal connections that link together the events of the story. Despite Wilson’s idea, therefore, the way in which the segments of discourse are causally connected plays an important role in the processing of global coherence.

However, there is more. To be in favor of the autonomy of coherence from relevance comes out something more than the reference to the causal structure of events alone; the fact that, as I have already pointed out, narrative has an inherently temporal structure. One of the main features of the holistic character of narrative is that the understanding of the gist of a story involves the connection between events distant in time. A consideration of this kind has important effects on cognitive architectures: The temporal character of narrative requires a kind of processing system that cannot be reduced to the function mode of mindreading. The cognitive system at the basis of relevance does not have the computational resources required to process the temporal level of storytelling (Ferretti and Adornetti, in press).

The first consideration is related to the automatic nature of mindreading processing. Apperly and Butterfill (2009) distinguished two different types of mindreading: the first (i.e., automatic, fast, and unconscious) emerges early in a child, while the second (i.e., deliberate, slow, and amenable to conscious introspection) emerges only later. In Scott-Phillips’ (2014) opinion, for the processing speed required, the mindreading involved in language must be the one of the first type. An important clue in favor of Scott-Phillips’ opinion is the fact that the recognition of the speaker’s intentions is a non-demanding task. That said, if the narrative processing was reducible to the reading of the speaker’s intentions alone, then the narrative plan would imply an automatic and effortless processing. Narrative understanding, however, as is evident from the experience of each of us, requires a considerable processing effort. The fact that narrative processing involves effort is an important clue against the idea that global coherence processing is managed by mindreading alone. Another important clue is the fact that the ability to grasp the speaker’s intentions is characterized as an immediate and punctuated process (i.e., unextended in space and time). Jolliffe and Baron-Cohen (2000) captured an important aspect of the matter by arguing that narrative processing cannot be referred to processes that, by their character of immediacy, can be processed within the confined space of the working memory. A consideration of this type has implications not only for defining the type of cognitive architectures involved in making narrative, but also defining the type of information processed. Following Apperly (2011), it is possible to attribute a quasi-perceptual character to the kind of information that is processed by the first type of mindreading. In addition, it is in reference to this kind of information that Scott-Phillips (2014, p. 72) maintained:

that mindreading is often less like thinking, and more like perception. (…) Certainly, many experiments have now showed that we track the beliefs of others automatically, as part of our intuitive monitoring of the world around us, and that like our perceptual experiences, these representations of others’ mental states fade quickly if we do not focus on them (…).

My opinion is that the quasi-perceptual character of mindreading makes this processing device unable to process the kind of information that characterizes narrative. The inherently temporal (which is also inherently spatial, as I shall see) nature of narrative leads us to suppose that mindreading is not the device that is able to ensure the human processing ability to tell stories. To process the temporal nature of narrative, first of all, what we need is a time machine.

### The Time Machine

The recruitment in Oldowan niche has brought to the fore the social role of the intellect. As noted above, however, the recruitment for cooperative purposes requires something more than the unique functioning of the social brain. Gärdenfors (2003) and Gärdenfors and Osvath (2010) showed that the cooperative foundation of human communication is closely related to the ability of individuals to project themselves into a space-time that is different from the current one. In Osvath and Gärdenfors (2005; Gärdenfors and Osvath, 2010) opinion, prospective cognition represents the adaptive answer to the selective pressures of Oldowan niche:

The Oldowan lifestyle was in a way signified by an extension of time and space. For example, there were long delays between the acquisition and the use of the tool, as well as considerable geographical distances between the sources of tool raw material sources and killing sites. (…) We submit that these cognitive skills became more important of the Oldowan hominins and had an obvious ecological use (Gärdenfors and Osvath, 2010, p. 108).

At present, we know that the ability to travel in time is tied to a specific cognitive system. Suddendorf and Corballis (1997, 2007) coined the term Mental Time Travel (MTT) to refer to “the faculty that allows humans to mentally project themselves backward in time to re-live, or forward to prelive events” (Suddendorf and Corballis, 2007, p. 299). The idea that MTT would be one of the cognitive systems at the foundation of human evolution is widely shared. That said, what is the relationship between the ability to travel in time and language? The prevailing view associates projection systems with the birth of symbolic thinking. At the base of Gärdenfors and Osvath’s (2010, P. 104) thesis is the idea that

the evolutionary gain of being able to communicate about referents that are not yet present is that collaborative forms of long-term planning become possible. Symbolic communication is an efficient way of solving problems concerning cooperation about future goals – more efficient than iconic miming.

As several authors argued that the distinguishing feature of human communication is its symbolic nature (Deacon, 1997), the question of whether the use of symbols is a prerogative of human communication is controversial (e.g., Seyfarth et al., 1980; Savage-Rumbaugh et al., 1986; Lyn et al., 2011). I would not explore in detail this dispute. The point of my interest is that, irrespective of the role that the prospective cognition has in respect to the origins of symbolic communication, the role of the ecological brain can be used in favor of a stronger and less controversial evidence of the distinctive character of human communication. My thesis is that through the extension in time and space imposed by the Oldowan niche, the prospective cognition has promoted a selective pressure for the development of the ability to tell stories. From my point of view, in fact, the cognitive systems that allow individuals to travel in space and time do not affect only the symbolic character of the communication but also the way in which symbolic expressions are organized in discourses governed by global coherence (Cosentino, 2011; Cosentino and Ferretti, 2014; Ferretti, 2014). Following Corballis (2011, p. 111), it is possible to maintain that “the same constructive process that allows us to reconstruct the past and construct possible futures also allows us to invent stories”. What is the empirical evidence to support the idea that the navigation systems in space and time are the basis of our ability to tell stories?

### Time, Space, and Narrative

If my hypothesis is correct, the ability to navigate in time must have repercussions on the narrative level and, in particular, on global coherences — the property that I have laid the foundation of human ability to build appropriate discourses. In order to evaluate this hypothesis (in a study conducted with the University of Udine and the Bambino Gesù Hospital in Rome), we have submitted autistic children to a narrative test. At the basis of our research were two orders of considerations. The first concerned the data related to the projection in time capabilities of autistic subjects (e.g., Jackson and Atance, 2008; Lind and Bowler, 2010; Lind et al., 2014; Marini et al., 2016). The second concerned the results related to deficit of global coherence in the narrative of these subjects (cf. for a review Stirling et al., 2014). Research on MTT skills in autistic children and those related to global coherence deficits are independent and, to our knowledge, there has been no finalized research to identify a causal correlation between the difficulties of autistic children in time projection and their narrative skills. In the study we conducted, we hypothesized that the narrative deficits of these subjects would respond specifically to their inability to travel in time. In our research, we administered non-linguistic tasks of MTT to autistic children in order to determine a subgroup of subjects with deficit in time-traveling. The hypothesis was that this subgroup of individuals showed greater difficulties in building global coherent stories. The data from our research confirmed the hypothesis (Marini et al., submitted).

These results represent an important step in the re-evaluation of the MTT role in processing narrative. This is an important clarification with respect to the studies that considered global coherence in reference exclusive to the causal links between segments of discourse (as in Trabasso’s model): Global coherence is the joint product of the processing systems that manage the causal structure of discourse segments and the projection systems that manage the traveling (back and forward) into the plot of the story. Given the importance of the narrative capacity in defining the specific properties of human communication, the idea that the projection devices are in the foreground among of the systems that regulate global coherence is an important step in defining a language model very differently from that assumed by the proponents of the social brain.

So far, I have insisted on the temporal nature of the narrative and on the role played by the projection in time in processing storytelling. Since to claim that the projection in time has a role in managing the global coherence is, as we shall see, largely equivalent to claim that projection in space has a role in processing global coherence, it is time to pay attention to the space navigation devices. In *The Art of Memory*, Yates (1966) describes the “loci method” used by ancient Greek and Roman orators to maintain the route of discourse in public debates: This method makes extensive use of the metaphor of navigation and, in particular, the construction of specific spaces located along the route whose attractiveness plays a central role in the construction of the discourse plan. In effect, spatial navigation represents, even intuitively, a good metaphor for thinking about the processes at the foundation of discourse and narrative. Both Lewis’ definition (1994, p. 82) that stated “the first requirements of any system of navigation is to enable the voyager to take his departure and continue toward his objective in the right direction” and Gallistel’s (1990, p. 35) idea that navigation is “the process of determining and maintaining a course or trajectory from one place to another” illustrate perfectly my perspective. The ability to maintain a trajectory in the right direction is a core component of the process involved in approaching a destination. Indeed, in order to reach the expected destination, one needs to keep the intended route and overcome geographic obstacles. In a very similar way, the process of discourse construction also relies on the ability to identify a goal (i.e., the content the speaker intends to convey to the listener) and to construct the route and maintain the right trajectory to express it. Similar to navigation in space, the discourse plan is strongly linked to difficulties in maintaining the course to reach a given destination. In fact, in the same manner as in space navigation, the achievement of the communicative goal depends on the continuous realignments implemented by speakers to rebuild the route in the face of continual digressions imposed by the different points of view that are typical of verbal communication (Ferretti, 2013; Ferretti et al., 2013). Building the route and maintaining the right trajectory to the goal is equivalent, in narrative terms, to building and maintaining the global coherence of discourse. What kind of evidence can we offer to justify the involvement of navigation systems in the processing of discourse coherence? Starting from the empirical evidence of narrative deficits in individuals with Williams syndrome (WS) (Marini et al., 2010) and the difficulties of these subjects in spatial orientation and navigation (e.g., Lakusta et al., 2010) in Ferretti et al. (2013), we assumed that the narrative difficulties shown by WS subjects could refer to their difficulties in navigation in space. Although at the moment there are not direct experimental results (e.g., as in the case of autism) to support my assumption, the idea that the difficulty of navigation in space of WS subjects could be related to their narrative difficulties is not only a speculative hypothesis. In support of my thesis I can appeal to two forms of empirical data. The first concerns the fact that space and time are based on (at least in some of its fundamental constituents) a widely shared neural substrate. The second concerns the experimental data on the role of the hippocampus (i.e., the shared neural substrate) in the ability to tell stories.

At the level of ordinary language, the use of spatial metaphors to conceptualize time (as when we say that future in front of us) is a well-known phenomenon (Lakoff and Johnson, 1980; for a recent review of the huge debate on the topic Athanasopoulos et al., 2016). A phenomenon of this kind seems warranted by the fact that the close link between space and time representation is well demonstrated by brain anatomy (Corballis, 2013, 2015). The discovery of place cells has allowed O’Keefe and Nadel (1978) to argue that the hippocampus, that is the basis of spatial cognition in rodents, is the substratum for episodic memory of humans (Dudchenko, 2010). The fact that the hippocampus is behind much of the construction of both our spatial maps and our mental journeys backward and forward in time is well known and uncontroversial (Corballis, 2015). However, are there any empirical data in favor of the thesis that the hippocampus is also involved in storytelling tasks?

MacKay et al. (1998) investigated the role of the hippocampus in managing global coherence by providing data in favor of the specific role of binding that this brain system plays in the discourse-level integration. While MacKay et al. (1998) examined only the role of past projections, the study of Race et al. (2015, p. 279) is the first to have tested the role of the hippocampus in storytelling by considering also the projections into the future. Using amnesic patients with Medial Temporal Lobe lesions, Race et al. (2015, p. 279) proved the role of the hippocampus in the construction of linguistically cohesive and coherent discourses. Of particular interest to my purposes is the definition of global coherence proposed by the authors at the conclusion of their work. After maintaining that “contextual measures of discourse coherence reflect the degree to which a narrative is oriented in space and time” and that “the hippocampus supports the creation of narrative context by structuring linguistic elements around spatiotemporally specific details”, Race et al. (2015, p. 279) proposed a definition of global coherence perfectly in line with my hypothesis in this article: “Discourse coherence reflects the development of a unified theme, spatiotemporally specific context, and chronologically ordered narrative”.

The thesis of the space-time basis of narrative ability seems to be empirically as well as theoretically justified. The result of the arguments summarized to this point is that the ecological brain plays an important role in the actual processing of the narrative language. Nevertheless, the justification of the role of projection systems in narrative processing is still not a proof of the fact that the ecological brain is involved in the origin of language. Therefore, the remaining step to take is to prove that the narrative skills represent the evolutionary drive to the evolution of human communication.

## The Narrative Origin of Language

The intuitive thesis of the relationship between language and narrative is that the ability to tell stories represents the final outcome of an evolutionary process that has in language its starting point. To argue that narrative skills are the core abilities required for the origin of language is to justify a strongly counterintuitive hypothesis. However, how do we justify this hypothesis? The first step in support of the narrative origin of language thesis is to show the possibility of a form of storytelling without language. While the thesis that narrative must be considered the product of language is supported by distinguished scholars (Scalise Sugiyama, 2005; Collins, 2013), it is equally possible to argue that storytelling is independent from language (for a review, cf. Sibierska, in press). Boyd (2009, p. 159), for example, has suggested that “narrative need not involve language. It can operate through modes like mime, still pictures, shadow-puppets, or silent movies. It need not be *restricted* to language”. The idea that narrative is independent from language supports the thesis that our ability to tell stories may pre-date language, and could potentially have underpinned the latter’s evolution. The statement that narrative is language-independent paves the way for the hypothesis that our ancestral relatives were able to tell stories before they started talking. Specifically, the acknowledgment that our ancestors possessed cognitive systems responsible for space-time projections suggests that they were endowed with the cognitive tools necessary to manage narrative representations long before (and independently from) their ability to manage language. By what means, then, did the transition from the ability to produce narrative representations to the narrative origin of language come about? The argument that binds the narrative form of representation to the origin of language is based on the theoretical assumption that thought, which precedes language, imposes a constraint upon the form of thought’s communication (cf. Cosentino and Ferretti, 2015). From this assumption, it follows that, if humans think in a predominantly narrative form, it is plausible to maintain that language (whose primary, but not exclusive, function is to express thoughts) finds the most appropriate form for its expression in the narrative medium. Following Corballis (2015, 2016), the origin of language should be considered an event driven by the need to make public the individual (internal and private) mental projections in space and in time. If the form of representation imposes constraints on the way we communicate our thoughts, this means that our ancestors were once faced with the requirement to invent a proper communicative medium to express the mental narratives they used to represent reality. Pantomime was thus the ideal communicative medium used by our ancestors to express the spatial-temporal nature of this representation. As such, it may be assumed that this is the basis on which our linguistic narrative abilities are founded.

From the communicative point of view, the advantage of pantomime is that, as pointed out by Arbib (2012), quoting Stokoe (2001), “provides open-ended possibilities for communication that work without prior instruction of convention” (Arbib, 2012, p. 219). Pantomime, in fact, represents the connection ring between lower level non-conventional capabilities such as imitation and higher level conventional capabilities such the proto-sign. Pantomimic communication is easy to understand (the iconic character gives it transparency), and its functioning does not require specifically communicative processing devices. Such considerations militate in favor of the idea that, by using pantomime, our ancestral relatives were able to tell stories long before (and in a total absence of) language. As McBride (2014, p. 3) maintained:

Mimes are not language. The proposal is that mimes come into being as a way of telling stories long before any possibility of language existed or was even anticipated. Mime was a complete storytelling process well within the talents of the hominins in whose bands it occurred. These individuals had zero concept of language, but they could manage mimed stories and understand them. Mimes and their understanding required nothing that every hominin did not already have.

The arguments in favor of the autonomous and independent nature of pantomime mark a point in favor of the idea that the ability to tell stories can be considered a skill that our ancient precursors possessed long before they were able to use a language. However, recognizing the autonomous and independent character of pantomime is not enough to explain how it can be considered as the evolutionary impulse that gave rise to human language. In order to gain this result, there remain few issues to address.

A first step is the definition of pantomime. Zywiczynski et al. (in press) in a review article proposed a very articulated taxonomy. While I recognize the importance of a detailed distinction of pantomime for the study of the origin of language, in this paper, I discuss it from a more general point of view: the analysis of the theoretical approaches that focus on those distinguishing aspects of pantomime from symbols. According to the proponents of the bodily mimesis hypothesis (Donald, 1991, 2001; Zlatev, 2008, 2014) for example, pantomime represents the bridge to study the transition to symbolic communication. Donald (1991, 2001) is the founder of a view that considers mimetic culture (of which pantomime is an integral part) as the evolutionary precursor of symbolic culture. According to these authors, the selective pressures driving the transition from iconic gestures toward abstract and arbitrary symbols depend on the difficulties of pantomime in representing events or objects. In Arbib’s (2012, p. 219) opinion “it’s hard to pantomime blue”; also Corballis (2015, p. 91) insisted on the same point: “[P]antomime is inefficient and often ambiguous, and needed to be developed into a system of symbols whose meanings were clear, and understood by members of the community”. Enhancing the difficulty of pantomime (focusing on its negative traits) is a way to explain how pantomime could have acted as a selective pressure directed to the birth of an arbitrary and abstract expressive system.

That said, there is a different scenario to consider. The role of pantomime in narrative paves the way to the opportunity to take into account a positive role of pantomime in the origin of language. In effect, even if pantomime is marked by ambiguity and inefficiency in representing the details of objects and events, pantomime also permits the inclusion of objects and events in a uniform and coherent frame of reference. So interpreted, pantomime represents the expressive tool that is able to bridge together the narrative character of human representation of reality with the narrative character of human communication of reality. From this point of view, enhancing the positive role of pantomime in language means enhancing those aspects of communication without which human language would not function properly, and above all, it would never have originated.

## Conclusion

In the *pars destruens* of this article, I have examined the view of the social brain hypothesis of the origin of language. Against this hypothesis, my idea is that the models inspired by the primacy of the social brain are not able to draw a useful distinction between animal and human communication. If the social brain is not enough to justify the distinctive character of human language, then, at best, the social brain is a necessary but not sufficient condition to explain the origin of human language.

Against the primacy of the social brain, in the *pars construens* of this article, I argued that human language has a narrative foundation, and that a pivotal role in the evolution of those abilities that allowed humans to free themselves from the mechanical automation of animal communication has been played by navigational systems in space and time. Such a conception of language opens the way to the idea that the ecological brain has a role as much important as the social brain in explaining for language origin. In addition to explaining the evolution of the symbolic level of language, the navigational systems in space and time are able to explain how humans can process narrative representations of reality and forms of narrative communication of reality.

Without the ecological brain at their disposal, our ancestral relatives could never tell stories; without being able to tell stories, as should be clear at this point, they would have never developed the kind of communication that distinguishes humans from all other animals.

## Author Contributions

The author confirms being the sole contributor of this work and approved it for publication.

## Conflict of Interest Statement

The author declares that the research was conducted in the absence of any commercial or financial relationships that could be construed as a potential conflict of interest.
